# Stone Impacted in the Lower End of the Ureter

**Published:** 1916-09

**Authors:** Courtney W. Shropshire, Chas. Waterson

**Affiliations:** Visiting Genito-Urinary Surgeon to the Hillman and Saint Vincents Hospitals, Birmingham; Consulting Urologist to the Birmingham Infirmary; Birmingham, Ala.


### STONES IMPACTED IN THE LOWER END OF THE URETER.

By Courtney W. Shropshire, M. D., Visiting Genito-Urinary Surgeon to the Hillman and Saint Vincents Hospitals, Birmingham; Consulting Urologist to the Birmingham Infirmary, and Chas. Watterston, M. D., Birmingham, Ala.

Advanced methods of diagnosis have within the last few years reduced our failures to a minimum. Especially is this true of surgery of the urinary tract, where thanks to the efforts of the cystoscopist and roentgenologist, the error has been reduced to practically zero.

Symptoms of renal colic, such as pain, haematuria, fre-

Read before the Chattahoochee Valley Medical and Surgical Association, Alexander City, July 11-13, 1916.

quent desire to urinate, and nausea are simply indications for a thorough cystoscopic and radiographic examination of the urinary tract and cannot be relied upon by the physician in reaching a conclusion in the case.

In the upper right quadrant gall stones would confuse the diagnosis, while in the right inguinal region lesions of the appendix would have to be considered. In the male seminal vesiculitis with vesicular colic would mislead the surgeon, and in the female numerous lesions of the pelvic organs would make a combined cystoscopic and x-ray examination a necessity.

It is our practice to do a functional kidney test, and pass shadow catheters to the pelvis of the kidney, or as high as possible, in every case of suspected stone, and though it seems a useless repetition, we wish to say that radiographic plates of the urinary tract, without the aid of the shadow catheter, for the purpose of locating stone, are useless, and sometimes worse than useless, for they give the surgeon a false sense of security in the diagnosis.

To facilitate the classification of the location of stone, in the ureter we may divide this structure into four parts:

First: The ureteropelvic junction.
Second: Upper third.
Third: Middle third.
Fourth: Lower third.

Judging from our records and the literature of the last three years, the location of ureteral stone would be as follows:

| | |
|---|---|
| Ureteropelvic junction | 11.5 per cent. |
| Upper third | 13. per cent. |
| Middle third | 1. per cent. |
| Lower third | 74.5 per cent. |

It is interesting to note the chemical composition as well as the size of stones, and see what effect size has on the character of the symptoms.

Watson, of Boston, after studying the chemical composition of one hundred and twenty renal and ureteral calculi reported the chemical composition as follows: Oxalate, 34 per cent;

Uric acid, 24 per cent; Mixed, 16 per cent.; Phosphatic, 22 per cent.; Cystine, 4 per cent.

This report is essentially the same as reports presented by other authors and, probably represents as nearly as we are able to ascertain the true relationship existing between stone from a chemical view.

A dense shadow is cast by a stone containing a large amount of oxalate and this is not surprising in view of the fact that calcium deposits in the veins and glands also cause a very dense shadow.

Uric acid stone is the most difficult to demonstrate in the x-ray plate, but with proper technique it can be shown in every instance. In the event that there is any doubt regarding the identity of a shadow it is best to inject the pelvis of the kidney and ureter with thorium or some silver salt to show its exact relationship with the ureter.

Since a majority of the ureteral stones are located in the lower third of the ureter, it seems but reasonable to call attention to the fact, that great care must be taken in making radiographic plates, for the density of the boney pelvis must be considered.

The size of the stone has little or nothing to do with the degree of pain for recently Burger reported the removal of two calculi one 17x9 m. m. and the other two and one-eighth by one and one-eighth inches with vague symptoms, and the diagnosis was made only by means of combined cystoscopic and x-ray examination. This is accounted for by the fact that a calculus sets up considerable irritation with resulting dilation of the ureter at the point of obstruction which is accompanied by thickening of the ureteral wall and periureteritis. It was even possible in one of Burger's cases to pass a number five catheter beyond the point of location of the stone. After this point was passed a sudden gush of urine ensued indicating a pelvic retention.

Very small stones will sometimes cause the most acute symptoms due probably to a sudden blocking of the ureter with a resulting distention of the renal pelvis.

The passage of a stone through the ureter according to Bevan causes no pain and he is no doubt correct in this statement as proven: First: By the occurence of renal colic in kink. Second: By renal colic when the ureter is occluded by blood clots. Third: Because drainage of the renal pelvis through an external wound relieves renal colic. Fourth: Because the passage of a catheter is not attended with pain. Fifth: Because over distention of the renal pelvis with any solution injected through a catheter causes typical renal colic.

It is therefore imposible to tell anything regarding the exact location of a stone by the character of the patient's symptoms or the location of the patient's pain.

Recently through the efforts of Doctor Hinman the waxed tipped catheter as used by Kelly is being more generally employed, but it is difficult to handle in the male subject and gives no better results than the combined cystoscopic and radiographic examination.

Treatment of ureteral calculus is divided into: Expectant. Operative. Cysctoscopic.

Expectant treatment is to be considered in all cases, where it is shown that the stone is of such size, that it is possible for it to pass through the ureter, for in a great many instances the stone will be forced into the bladder and expelled during the act of urination if given sufficient time.

During the period of expectancy it is well to give large quantities of water and in practically every case it is necessary to give morphine to relieve the pain.

The attack will pass off after a variable length of time, the pain being relieved by the passage of the stone into the bladder, or an increase in the size of the ureter, at the point of impaction. The patient should be instructed to strain the urine through a cloth to enable the physician or surgeon in charge of the case to tell whether the stone has passed or not.

It is best to combine the expectant treatment with the systoscopic, for by doing this we increase greatly the chance of relieving the patient.

Braasch has reported two hundred and ninety-four case in sixty of which the stone was removed during cystoscopic examination or passed immediately afterward.

It is not surprising that efforts have been made to remove stones impacted in the ureter by means of the cystoscope, and especially so since it has been demonstrated that a majority of the stones are located in the lower part of this structure, where open operation is most difficult.

Lewis in 1912 devised a very satisfactory method of dealing with stone by means of the Bransford Lewis operating cystoscope together with scissors, dilators, and forceps for use with his instrument, and in the same article he reported several cases of successful removal.

Burger writing in the American Journal of Surgery in the same year described a method of dilating the ureter through a Brown-Burger cystoscope by means of olivary bougie with the aid of the D'Asonval current. This method is painless and gives very satisfactory results.

The following equipment is necessary: Operating cystoscope, High frequency machine giving D'Asonval current; Insulated wire with screw point for carrying olivary tips.

It is very easy to dilate the ureter from the size of a number six catheter to the size of a number twelve French sound or to a fourteen if necessary. The operation takes but a few minutes, and a large well lubricated dilator can then be introduced in an effort to dislodge the stone and change its position so as to favor descent. It is customary after this procedure to inject olive oil or glycerine to facilitate the downward passage of the stone; however, this is of doubtful value.

The following case reports illustrate this method of treatment:

Case No. 1. E. B. White, male age 40, occupation laborer. Admitted to the Hillman Hospital, June 12th, 1915. Complaint, Dull pain in the back and vesical irritability. Has had several attacks of renal colic and passed a small stone two years ago. Examination of the urine was negative.

Cystoscopic examination June 13th, revealed a bulging in the muccous membrane of the bladder about two m. m. above the os. It was impossible to pass a catheter beyond this point. Indigo-carmine right kidney nine minutes, left kidney eleven minutes. Radiographic plate with shadow catheter to point of obstruction on left and to kidney pelvis on right shows a small shadow, probably a calculus, in relation with the catheter on the left side.

Ureteral os would admit only a number five ureteral catheter. Patient was brought to my office the following day and the ureter dilated up to a number twelve French, in the course of fifteen minutes. Following a large ureteral dilator was introduced and the stone was dislodged. Twenty minutes after the dilator was removed the stone passed into the bladder and was picked up with a Burger forcep.

This stone was so low down in the ureter it could be plainly seen bulging through the mucous membrane of the bladder.

Case No. 11. Dr. W. L. R. White, male, age 42. Has had several attacks of renal colic the last being the day previous to this examination. The patient is very sore and tender in the region of the left kidney and his slight rigidity of left rectus. Cystoscopic examination as follows:

Bladder normal, right ureter normal. Left ureter congested. Catheter passed to kidney, on right side, on left side obstruction is met with about five m. m. above the os. Indigo-carmine right side ten minutes, left side fifteen minutes. Shadow catheters passed and radiographic plates made. Small shadow on left side corresponding to point of obstruction.

Left ureter dilated with D'Arsonval current and about five c. c. of sterile olive oil injected. Stone was passed per uretha the following day. This stone was about the size of a grain of wheat. The patient was completely relieved and has had no trouble since.

The operative treatment would be considered last and should be resorted to only in the event that other measures fail. To expose the lower end of the ureter the incision described by Charles Gigson is the most satisfactory. It begins in the medium line just above the pubice follows the curve of pouparts ligament outward and turns upward just inside the anterior superior spine. It is often necessary to make another incision at right angles to the first along the outer border of the rectus. The flap is dissected up and good exposure is obtained.

This is an extensive operation and as was said earlier in this paper it is not to be resorted to unless our other efforts are unavailing.

In conclusion we wish to say:

First: That a majority of the ureteral stones are located in the lower third of the ureter.

Second: Combined cystoscopic and x-ray examination are necessary in making a diagnosis.

Third: Renal colic follows occlusion of the ureter and is due to over distention of the renal pelvis and not to the presence of a stone in the ureter.

Fourth: The cystoscopic or trans-vesical removal of stone in the ureter is the method of selection in the treatment of this condition.

### Literature.

Stones in The Ureter, William F. Braasch, M. D., and Alexander B. Moore, M. D., Jour. A. M. A., Oct. 9, 1915, pp. 1234-1237.

Report of 110 Cases of Renal and Ureteral Calculi and of Ten Cases Simulating These Conditions With Comments. F. S. Watson, M. D., Boston Med. & Surg. Jour., Jan. 9, 1913, pp. 37-43.

The Diagnosis of Stone in the Pelvic Portion of the Ureter. Hugh Cabot, M. D., and W. J. Dood, Boston Med. & Surg. Jour., July 21, 1910, pp. 85-89.

Unusually Large Ureteral Calculi. Leo. Burger, M. D. New York Med. Jour., Dec. 5, 1914, pp. 1103-1108.

Kidney and Ureteral Stone. Arthur Dean Bevan, M. D. Jour. A. M. A., Feb. 26, 1910, pp. 665-670.

The Diagnosis and Treatment of Renal Calculi. Joseph F. Smith, M. D. Wisconsin Med. Jour., Oct., 1909, pp. 224-235.

The Diagnosis and Treatment of Stone in the Ureter. Arthur Dean Bevan, M. D., and Herman L. Kretschumer, M. D. Illinois Med. Jour., Nov. 1909, pp. 1143-1148.

A New Method of Facilitating the Passage of Descending Ureteral Calculi. Leo. Burger, M. D. American Jour. of Surgery, Jan. 1912, pp. 1-7.

The Removal of Ureteral Stone by Bystoscopic Methods. Bransford Lewis, M. D. New York Med. Jour., Nov. 16, 1912, pp. 879-902. 327-328-329 First National Bank Building.

